# Molecular docking and molecular dynamics simulation studies of inhibitor candidates against *Anopheles gambiae* 3-hydroxykynurenine transaminase and implications on vector control

**DOI:** 10.1016/j.heliyon.2025.e41633

**Published:** 2025-01-02

**Authors:** Eunice O. Adedeji, Olubanke O. Ogunlana, Gbolahan O. Oduselu, Rainer Koenig, Ezekiel Adebiyi, Opeyemi S. Soremekun, Segun Fatumo

**Affiliations:** a Covenant University Bioinformatics Research (CUBRe), Covenant University, Ota, Ogun State, Nigeria; b Department of Biochemistry, Covenant University, Ota, Ogun State, Nigeria; c Department of Biology, University of York, York, United Kingdom; d Covenant Applied Informatics and Communication Africa Centre of Excellence, Covenant University, Ota, Nigeria; e Institute for Infectious Diseases and Infection Control (IIMK, RG Systems Biology), Jena University Hospital, Am Klinikum 1, 07747, Jena, Germany; f African Center of Excellence in Bioinformatics & Data Intensive Science, Makerere University, 10218, Kampala, Uganda; g Division of Applied Bioinformatics, German Cancer Research Center (DKFZ), G200, Im Neuenheimer Feld 280, 69120, Heidelberg, Germany; h The African Computational Genomics (TACG) Research Group, MRC/UVRI, and LSHTM, Entebbe, Uganda; i Precision Healthcare University Research Institute, Queen Mary University of London, United Kingdom; j Department of Non-Communicable Disease Epidemiology, London School of Hygiene & Tropical Medicine, United Kingdom

**Keywords:** 1,2,4-Oxadiazole, 3HKT, Isoxazoles, Larvicide, Mosquito, Vector control

## Abstract

Isoxazole and oxadiazole derivatives inhibiting 3-hydroxykynurenine transaminase (3HKT) are potential larvicidal candidates. This study aims to identify more suited potential inhibitors of *Anopheles gambiae* 3HKT (*Ag*3HKT) through molecular docking and molecular dynamics simulation. A total of 958 compounds were docked against *Anopheles gambiae* 3HKT (PDB ID: 2CH2) using Autodock vina and Autodock4. The top three identified hits were subjected to 300 ns molecular dynamics simulation using AMBER 18 and ADMET analysis using SWISSADME predictor and ADMETSAR. Replacement of alkyl attachment on C5 of isoxazole or oxadiazole derivative with a cycloalkyl group yielded compounds with lower binding energy than their straight chain counterparts. The top three compounds were brominated compounds, 2‐[3‐(4‐bromophenyl)‐1,2‐oxazol‐5‐yl]cyclopentane‐1‐carboxylic acid, 2-[3-(4-bromophenyl)-1,2,4-oxadiazol-5-yl]cyclopentane-1-carboxylic acid, 3-[3-(4-bromo-2-methylphenyl)-1,2,4-oxadiazol-5-yl]cyclopentane-1-carboxylic acid, and they had binding energies of −8.58, −8.25, and −8.18 kcal/mol in virtual screening against 2CH2 protein target, respectively. These compounds were predicted to be less toxic than temephos, a standard larvicide and more biodegradable than previously reported inhibitors. The three compounds exhibited a greater stabilizing effect on 2CH2 protein target than 4-[3-(4-bromophenyl)-1,2,4-oxadiazol-5-yl]butanoic acid, a previously reported inhibitor candidate with good larvicidal activity on *Aedes aegypti*. Further thermodynamic calculations revealed that the top three compounds possessed total binding energies (ΔG_bind_) of −26.64 kcal/mol, −24.26 kcal/mol and −14.11 kcal/mol, respectively, as compared to −12.02 kcal/mol for 4-[3-(4-bromophenyl)-1,2,4-oxadiazol-5-yl]butanoic acid. These compounds could be better larvicides than previously reported isoxazole or oxadiazole derivatives and safer than temephos.

## 1 Introduction

Malaria is a global health challenge with over 200 million cases reported yearly between 2010 and 2022 and its notable footprint observed in sub-Saharan Africa [1]. These consistently high values of reported malaria cases clearly reveal the prevailing high transmission rate of the disease. Malaria is caused by *Plasmodium* spp and transmitted by female *Anopheles* mosquito [2]*.* Although about four hundred and sixty-five (465) species of *Anopheles* exist, only about forty-one (41) transmit malaria [3]. Among these vectors, *Anopheles gambiae* has been identified as the most effective vector for *Plasmodium falciparum* causing the deadliest form of Malaria [4]. Efforts have been made towards controlling these vectors through the use of insecticides for either adulticides or larvicides, thus preventing malaria transmission [5]. Unfortunately, some of these insecticides are toxic to non-target species since they target proteins that are also present in non-target species [6]. Likewise, *Anopheles gambiae* has significantly developed resistance to major classes of insecticides used in malaria control programs - organophosphates, carbamates, organochlorides and pyrethroids [[7], [8], [9], [10], [11]]. This resistance is due to the extensive use of a limited number of insecticide classes for vector control, leading to selection pressure [12]. Consequently, the development of new insecticides with different modes of action is necessary to provide more classes of compounds that can be rotated for vector control, thus preventing the development of resistance [13]. However, the success of this process is hinged on the identification of new targets and prospective inhibitors.

**Fig. 1:**
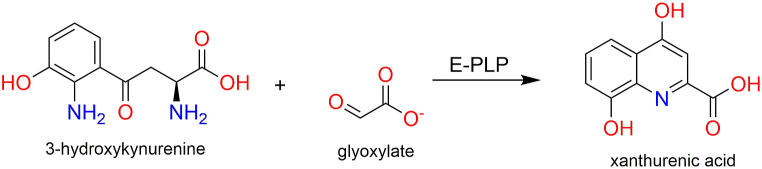
Enzyme function of 3-hydroxykynurenine transaminase (3HKT). *Ag*-HKT catalyzes the pyridoxal 5′-phosphate-dependent transamination of 3-hydroxykynurenine (3-HK) to xanthurenic acid (XA) using alpha-ketoacid glyoxylate as the preferred amino group acceptor.

Inhibitors of 3-hydroxykynurenine transaminase (3HKT) are gaining attention as potent compounds for vector control [14,15]. 3HKT has been identified as a potential target for insecticide development or transmission-blocking strategies [16]. It has also been predicted by choke point analysis to be an essential enzyme, consequently, a candidate for insecticide development [17]. It is a pyridoxal phosphate (PLP)-dependent enzyme that efficiently metabolizes 3-hydroxykynurenine (3HK) using glyoxylate to yield Xanthurenic acid (XA) (Fig. 1). The 3HK, which is the major substrate of this enzyme, is an important toxic metabolite of the tryptophan degradation via kynurenine pathway into XA [18]. It generates free radicals and results in neuronal death. Hence, the metabolism of 3HK is important to prevent toxicity and damage [19]. In humans, 3HK can be metabolized either by kynureninase or kynurenine aminotransferase (KAT) to yield 3-hydroxyanthranilic acid or xanthurenic acid respectively [20]. However, in organisms like *Anopheles* and *Aedes,* 3HK conversion to XA is the only detoxification route for 3HK [21]. While this reaction is catalyzed by KAT in humans, in *Anopheles* and *Aedes aegypti*, it is catalyzed by a specific 3HKT. This makes 3HKT a crucial enzyme in *Anopheles,* to prevent accumulation of toxic 3HK, and an attractive target for insecticide development. Studies have been carried out to investigate the structure, expression levels, biochemical activity, and importance of 3KHT. For instance, 3HKT in *An. gambiae* (*Ag*3HKT) has been identified, expressed and its biochemical activity determined [16]. The authors observed that 3HKT is highly expressed in larvae and adult mosquitoes, which reveals its importance in preventing toxicity damage to these stages of the mosquito. However, expression of 3HKT was low or absent in pupae, and this is explained by the necessity of 3HK accumulation for eye development that occurs in the pupae stage [16,22].

The 3-dimensional (3D) structures of *An. gambiae* in its PLP form (PDB ID: 2CH1) and in complex with 4-(2-aminophenyl)-4-oxobutyric acid, a competitive inhibitor (PDB ID: 2CH2) by x-ray crystallography have been determined [23]. The availability of these structures makes *in silico* screening of compounds as likely inhibitors of 3HKT through molecular docking and molecular dynamics simulation possible. Similarly, the availability of recombinant *Ag*3HKT, makes *in vitro* testing of promising inhibitor candidates possible. A LC-MS/MS based enzymatic assay to determine the kinetic constants values of recombinant *Ag*3HKT and evaluate its inhibition has been developed [24]. The authors tested 4-(2-aminophenyl)-4-oxobutyric acid and 4-(2-amino-3-hydroxyphenyl)-4-oxobutanoic acid on the setup and 4-(2-amino-3-hydroxyphenyl)-4-oxobutanoic acid was observed to be a more potent inhibitor of *Ag*3HKT. Taking a cue from the structure of 4-(2-aminophenyl)-4-oxobutyric acid, isoxazoles and 1,2,4-oxadiazole derivatives have been further synthesized and observed to inhibit 3HKT in *Ae. aegypti* with larvicidal activity [[25], [26], [27]].

Isoxazoles are five-membered heterocyclic compounds containing a nitrogen atom adjacent to an oxygen atom, while oxadiazoles are five-membered heterocyclic compounds with two nitrogen atoms and one oxygen atom. Several studies on the synthesis and larvicidal activities of these heterocycles have been reported. Da Silva-Alves et al. [25] examined the larvicidal activities of a series of 3,5-disubstituted isoxazoles, which were synthesized by the 1,3-dipolar cycloaddition of terminal alkynes to aryl nitrile oxides. They reported that the introduction of an isoxazole template instead of a 1,2,4-oxadiazole ring and the presence of electronegative substituents in the phenyl ring increased the larvicidal activities of the compounds [25]. The synthesis of isoxazole rings faces the challenge of ring opening, which leads to the production of *o*‐amino ketones. However, the introduction of transition‐metal catalysts has helped to mitigate this challenge [28]. Generally, isoxazole compounds have been reported to be better larvicidal compounds than 1,2,4-oxadiazole compounds [25,26]. Further to this, more soluble 1,2,4-oxadiazole derivatives have been synthesized, and their activities tested in *in vitro* and *in silico* studies [14,15]. Similarly, a QSAR studies revealed that 1,2,4-oxadiazole derivatives with bulkier substituents at the para position of the aromatic ring have better binding affinity to *Ae. aegypti* 3HKT [14]. The study further suggested that better oxadiazole-based inhibitors of 3HKT can be further developed. Recently, metadynamics (Meta-MD) simulations were conducted to determine the multidimensional free energy surface (FES) for the binding process between two HKT enzymes (*Ae*HKT, *Ag*HKT) and two inhibitors: 4OB (4-(2-aminophenyl)-4-oxobutyric acid), which was previously co-crystallized with *Ag*HKT, and a canonical representative of the 1,2,4-oxadiazole derivatives, sodium 4-(3-phenyl-1,2,4-oxadiazol-5-yl)butanoate (OXA) [29]. The findings from their study further revealed 1,2,4-oxadiazole derivatives also as potential larvicide candidates.

With the high transmission rates of malaria and the increasing insecticidal resistance in *Anopheles* to major insecticide classes, identifying suitable targets and potential inhibitors becomes significantly important for efficient vector control. Considering the benefits of hybrid virtual screening and molecular dynamic simulation in identifying suitable ligands of identified targets, this current study aims at carrying out a hybrid virtual screening and molecular dynamics simulation on *An. gambiae* 3HKT. This study retrieves and screens compounds (from PUBCHEM) structurally similar to previously reported 3HKT inhibitors based on 2D fingerprint-based Tanimoto coefficient. It further evaluates the interactions of these compounds with *Ag*3HKT through molecular docking and molecular dynamics simulation towards identifying more potential *Ag*3HKT inhibitors, which could be considered as larvicides. Implications of findings on malaria vector control were also presented. While this section presents a background to the study, section 2 presents the overall methodology adopted, section 3 presents the results obtained and section 4 discusses and concludes the work.

## 2 Methods

### 2.1 Retrieval and preparation of protein target

The 3D structure of *Ag*3HKT has been determined by x-ray crystallography [23] and is readily available in Research Collaboratory for Structural Bioinformatics (RCSB) protein data bank (PDB) [30]. The 3D structure of *Ag*3HKT complexed with a co-crystallized inhibitor, 4-(2-aminophenyl)-4-oxobutyric acid (KY1) was retrieved from RCSB PDB (PDB ID: 2CH2). The protein was prepared using Autodock Tools 1.5.7 [31]. The crystal structure of the protein contains 4 identical chains (A, B, C, D), with each chain having PLP bound (the enzyme is PLP-dependent) as well as its bound inhibitor, KY1. The four chains form two tight homodimers (A and D form one homodimer, while B and C form the second homodimer) [23]. Since a single homodimer is required for protein activity, inhibitors and one dimer of the protein (chains A and D) were removed, while PLP in chains B and C were retained. A single dimer containing chains B and C was used for further study. Further cleaning and preparation of the protein included removal of water molecules, computing of Gasteiger charges, addition of polar hydrogens and merging of non-polar hydrogens and conversion to pdbqt files [32].

### 2.2 Ligand preparation

Seven previously reported potential inhibitors of 3HKT were used as starting compounds to retrieve similar compounds. These inhibitors included 4-(2-aminophenyl)-4-oxobutyric acid (co-crystallized inhibitor of 2CH2) [23], 4-(2-Amino-3-hydroxyphenyl)-4-oxobutanoic acid [24], 4-[3-(4-bromophenyl)-1,2,4-oxadiazol-5-yl]butanoic acid, 3-(3-(4-Bromophenyl)-1,2,4-oxadiazol-5-yl)propanoic acid, 4-[3-(3-methylphenyl)-1,2,4-oxadiazol-5-yl]butanoic acid, 4-[3-(4-Iodophenyl)-1,2,4-oxadiazol-5-yl]butanoic acid, 4-(3-phenyl-1,2,4-oxadiazol-5-yl)butanoic acid [26]. Compounds similar to each of these potential inhibitors were retrieved from PubChem [33] using a Tanimoto threshold of 90 % and filtered based on Lipinski rule (−5:5[LOGP]) AND (0:500[MOLWT]) AND (0:5[HBDC]) AND (0:10[HBAC]) [34]. Only compounds which obeyed Lipinski rule were retrieved. A compound library having a total of 958 compounds was retrieved. The ligands were prepared by Universal Force Field (uff) energy minimization [35] and conversion to pdbqt files using the Open Babel tool in PyRx [36,37].

For evaluating the effect of cyclization on binding affinity, compounds were drawn using MarvinSketch 22.11 [38]. The alkyl side chain of some reported 1,2,4-oxadiazole derivatives was replaced with cyclic rings appropriately (cyclopropane-carboxylic for propanoic acids and cyclobutane-carboxylic acids for butanoic acids). In addition, respective isoxazole derivatives of these compounds (replacing the oxadiazole nucleus with an isoxazole nucleus) were drawn also. All compounds were prepared using Open Babel tool in PyRx.

### 2.3 Virtual screening

Autodock vina [39] was employed to carry out the virtual screening of 958 compounds. Further re-docking of the top 10 compounds from the virtual screening results, as well as screening of isoxazoles and cyclized derivatives of bromine compounds, was carried out using autodock4 in autodock tools 1.5.7. For the molecular docking, the grid box used had dimensions 42 × 44 × 52 Å for x, y, and z respectively and a spacing of 0.375 Å with grid centre x, y, z of −31.378, 7.896, 16.396 (for both autodock vina and autodock4). The grid box was prepared around previously reported active site residues - asparagine 44, serine 43 in chain B and arginine 356 and glycine 25 in chain C [23]. The Lamarckian genetic algorithm in autodock4 was employed [40]. The parameters specified for the algorithm included ten genetic algorithm runs, a population size of 150 individuals, maximum values of 27000 for generations and 2500000 for energy evaluations. Local search frequency parameters were also specified as 0.06 and the iteration limit for the local search was specified as 300. The crossover and gene mutation rates were specified as 0.8 and 0.02 respectively. Upon completion of the screening, binding energies were obtained, and Discovery Studio [41] was used to visualize the 2D binding interactions between compounds and *Ag*3HKT. The 3D positions of compounds in *Ag*3HKT were visualized in Chimera [42]. How strong the interaction of the ligands with the protein was established by how highly negative the binding energy was.

### 2.4 ADMET properties of ligands

The pharmacokinetic properties, drug-like nature and medicinal chemistry friendliness properties of the selected compounds were evaluated using SWISSADME predictor server [43], while eco-toxicity was predicted using ADMETSAR [44]. Parameters considered included permeability coefficient, Brenks filter, PAINS filter, aquatic toxicity, honey bee toxicity, biodegradability and synthetic accessibility score. The permeability coefficient, log Kp (cm/s), predicts the transport of compounds across human epidermis [45]. The more negative its value, the lesser the skin permeability of the compound. Brenks filter gives a structural alert of fragments in compounds that likely to be toxic and unstable. PAINS filter suggesting that these compounds might be target specific and not promiscuous binders. Synthetic accessibility score which ranges from 1 (very easy) to 10 (very difficult to synthetize) predicts the ease of synthesis.

### 2.5 Molecular dynamics study

The 3D structure of *An. gambiae* 3HKT (*Ag*3HKT) with PDB ID 2CH2 was retrieved from the Protein Data Bank [30]. Molecules co-crystallized with 3-hydroxykynurenine transaminase were deleted. MODELLER tools found in the graphic user interface of Chimera was employed to fill up missing residues [46]. The 2D structures of the ligands gotten via the virtual screening process were drawn using Marvin Sketch. While the structural optimization of the ligand was carried out using the UFF forcefield of Gaussian 16, the B3LYP/6–311 ++G was used for the geometrical optimization. Identification of the active site was carried out by centering the grid box around a co-crystallized ligand observed on the 3-hydroxykynurenine transaminase. While Chimera software was used for the molecular docking process [46], a simulation run of 300 ns was adopted for the system simulation using the Amber18 software [47].

The simulation was conducted using the FF18SB module of the AMBER force field, utilizing the General Amber Force Field (GAFF) and Restrained Electrostatic potential (RESP) to describe the atomic charges of the ligands. The system was neutralized, and hydrogen atoms were added using the Leap variant in Amber. Solvation was performed using an orthorhombic box of TIP3 water molecules placed at a distance of 9 Å from all *Ag*3HKT atoms. The resulting system was minimized in 2000 steps with a 500 kcal/mol restriction potential and then by an unrestrained full minimization of 1000 steps. The system was heated at −273.15 °C at 50 ps for simulation time while maintaining a 10 kcal/mol Å^−2^ harmonic restraint and a 1.0 ps^−1^collision frequency. After equilibration, the system was heated at 50 ps for each system while maintaining the number of atoms, a temperature of 26.85 °C, and a pressure of 1 bar. The hydrogen atom bonds were narrowed using the SNAKE algorithm with a simulation time of 300 ns for each algorithm. All steps of the simulation were run for 2 fs and kept at constant temperature and pressure using the SPFP precision model. Additional analysis like RMSD, RMSF, and Radius of Gyration were carried out using the PTRAJ variant of the Amber 14 and graphical plots were created using the ORIGIN analytical software tool and visualization was carried out using the UCSF Chimera software.

### 2.6 Thermodynamic estimations

The Molecular Mechanics/Poisson-Boltzmann Surface Area (MM/PBSA) [48] was used to estimate the binding strength between *Ag*3HKT and the ligands. MM/PBSA is described as an end-point energy estimation for calculating binding modes of ligands and their protein target. MM/PBSA is described as:

(1) ΔG_bind_ = G_complex_ - (G_receptor_ + G_inhibitor_)

(2) ΔG_bind_ = ΔG_gas_ + ΔG_sol_ - TΔS

(3) ΔG_gas_ = ΔE_int_ + ΔE_ele_ + ΔE_vdW_

(4) ΔG_sol_ = ΔG_ele,sol(PB)_ - ΔG_np,sol_

(5) ΔG_np,sol_ = γSASA + β

Where ΔG_gas_ = total gas-phase energy (estimated using intermolecular energy (ΔE_int_), electrostatic energy (ΔE_elel_) and van der Waals energy (ΔE_vdW_))

ΔG_sol_ = solvation energy
TΔS = entropy change
ΔG_ele,sol(PB)_ = polar desolvation energy
ΔG_np,sol_ = non-polar desolvation energy.
γ = surface tension proportionality constant (set to 0.0072 kcal/mol Å^−2^)
β is a constant equal to 0 and
SASA = solvent-accessible surface area (Å^2^).

## 3 Results

### 3.1 Virtual screening

**Fig. 2:**
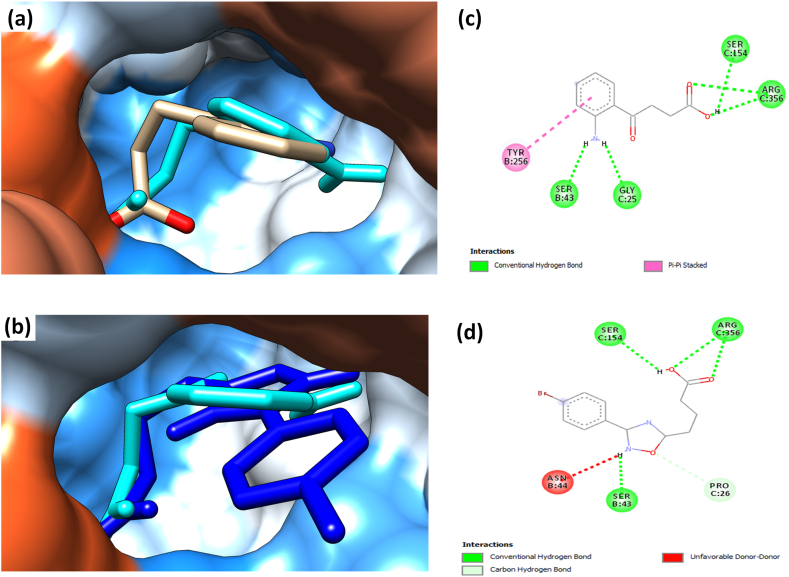
**(a)** 3D orientation of 4-(2-aminophenyl)-4-oxobutanoic acid (6420149) with the 3HKT docked using AutoDock Tools 1.7, the ligand represented in stick format in cyan, while co-crystallized inhibitor is light brown. **(b)** 3D orientation of 4-(2-aminophenyl)-4-oxobutanoic acid and 4-[3-(4-bromophenyl)-1,2,4-oxadiazol-5-yl]butanoic acid (17122279) docked with 3HKT using AutoDock Tools 1.7, the potential inhibitors are represented in stick format. 4-(2-aminophenyl)-4-oxobutanoic acid in cyan, 4-[3-(4-bromophenyl)-1,2,4-oxadiazol-5-yl]butanoic acid in deep blue **(c)** 2D interaction of 3HKT docked with 4-(2-aminophenyl)-4-oxobutanoic acid using AutoDock Tools 1.7, visualized in Discovery Studio: serine 43, glycine 25, serine 154 and arginine 356 form conventional hydrogen bonds represented in green dotted lines with the ligand. **(d)** 2D interaction of 3HKT docked with 4-[3-(4-bromophenyl)-1,2,4-oxadiazol-5-yl]butanoic acid (17122279): residue serine 43, serine 154 and arginine 356 form conventional hydrogen bonds represented in green dotted lines with the ligand.

To validate the applicability of virtual screening in guiding selection of hit compounds for insecticide development, the binding affinity of *Ag*3HKT (2CH2) for its co-crystallized ligand (6420149) and a previously reported inhibitor candidate of *Ae. aegypti* with good larvicidal activity (17122279 or 6d) was evaluated. The previously reported inhibitor candidate of *Ae. aegypti* (17122279) was considered because the HKT sequences of *Ae. aegypti* (*Ae*HKT) and *A. gambiae* (*Ag*HKT) share a 73 % identity, and the amino acid sequence in the active site of both enzymes is nearly identical, with the triad Ser43-Asn44-Phe45 involved in the substrate recognition process in both species [33]. 2CH2 protein target had a greater binding affinity for 17122279 (with binding energy of −7.38 kcal/mol and inhibitory constant of 3.91 μM) compared to the docked co-crystallized ligand, 6420149, (with binding energy of −6.17 kcal/mol and inhibitory constant of 30.15 μM). The docked co-crystallized ligand, 6420149 and previously reported inhibitor candidate, 17122279, interacted with the protein in the same binding pocket and in similar orientation with its co-crystallized form (Fig. 2 (a) and (b)). Further evaluation of the 2D interaction (Fig. 2 (c) and (d)) revealed that amino acid residues serine 43, serine 154 and arginine 356 in *Ag*3HKT form conventional hydrogen bonds with both ligands, which are active residues that have previously been reported.

From the 958 compounds screened, it was observed that compounds with a cycloalkyl-1-carboxylic acid replacement at the alkyl chain located at C-5 of 1,2,4-oxadiazole nucleus had greater binding affinity for *Ag*3HKT as depicted by the lower binding energies observed compared to their straight chain counterparts. The top compounds were cycloalkyl-1-carboxylic acid derivatives with bromine attachment (Table S1). The top ligands from the virtual screening differed from the co-crystallized ligand with binding energy >1.433 kcal/mol, suggesting that further cyclization might yield better inhibitors. Based on these results, the effect of replacing alkyl chains with cyclic rings as well as the effect of replacing with an isoxazole nucleus was further evaluated. With reference to the isoxazole and oxadiazole derivatives having bromine functional group on the C-4 of the phenyl ring, the isoxazole derivative with the cyclopentane‐1‐carboxylic acid attachment had the lowest binding energy (−8.58 kcal/mol), signifying a better affinity (Table 1), while the highest binding energy was observed for 1,2,4-oxadiazol-5-yl-propanoic derivative (−6.38 kcal/mol). These two ligands differed in binding energy by 2.2 kcal/mol, which might suggest the cyclopentane‐1‐carboxylic acid derivative to be a better *Ag*3HKT inhibitor candidate and potential larvicidal compound. The cyclopentane‐1‐carboxylic acid oxadiazole or isoxazole derivatives had lower binding energies than those with cyclobutane or cyclopropane attachments. This suggests that isoxazole or oxadiazole derivates with a cyclopentane chain as better larvicidal candidates than their cyclopropane and cyclobutane counterparts. Similarly, all derivatives with a cycloalkyl chain had lower binding energy than their straight chain alkyl counterparts (Table 1).

**Table 1:** Binding energies of the isoxazole and oxadiazole derivates with bromine attachment, when docked against the *Ag*3HKT protein target (PDB ID: 2CH2).

Name	Structure	Binding Energy *(kcal/mol)*
2-[3-(4-bromophenyl)-1,2-oxazol-5-yl]cyclopentane-1-carboxylic acid		−8.58
2-[3-(4-bromophenyl)-1,2-oxazol-5-yl]cyclobutane‐1-carboxylic acid		−8.4
2-[3-(4-bromophenyl)-1,2,4-oxadiazol-5-yl]cyclopentane-1-carboxylic acid		−8.25
2-[3-(4-bromophenyl)-1,2,4-oxadiazol-5-yl]cyclobutane-1-carboxylic acid		−7.81
4-[3-(4-bromophenyl)-1,2-oxazol-5-yl]butanoate		−7.8
2-[3-(4-bromophenyl)-1,2‐oxazol‐5‐yl]cyclopropane‐1‐carboxylate		−7.53
4-(3-(4-bromophenyl)-1,2,4-oxadiazol-5-yl)butanoic acid		−7.38
2-[3-(4-bromophenyl)-1,2,4-oxadiazol-5-yl]cyclopropane-1-carboxylic acid		−6.87
3-[3-(4-bromophenyl)-1,2‐oxazol‐5‐yl]propanoic acid		−6.61
3-(3-(4-bromophenyl)-1,2,4-oxadiazol-5-yl)propanoic acid		−6.38

**Fig. 3:**
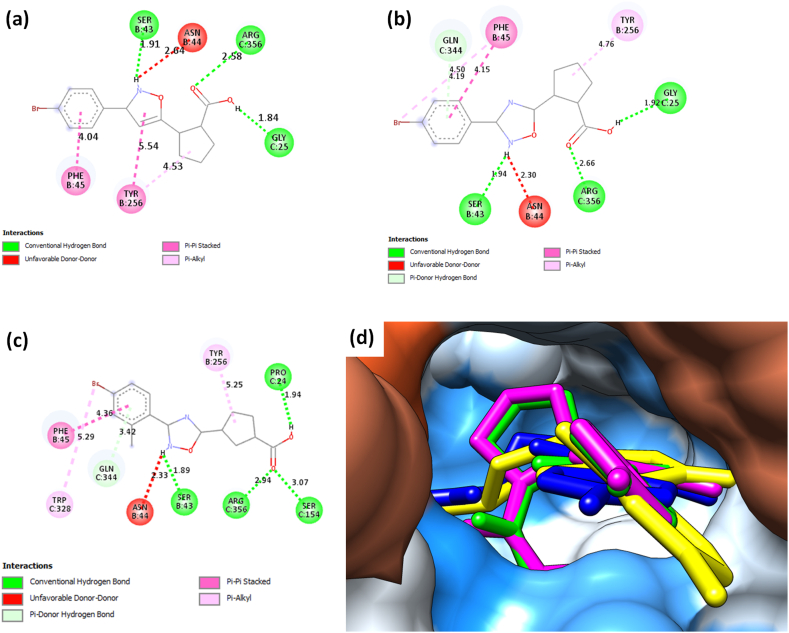
**(a)** 2D interaction of 3HKT docked with 2‐[3‐(4‐bromophenyl)‐1,2‐oxazol‐5‐yl]cyclopentane‐1‐carboxylic acid (Cmpd1) using AutoDock Tools 1.7, visualized in Discovery Studio: residue serine 43, glycine 25 and arginine 356 form conventional hydrogen bonds represented in green dotted lines with the ligand. **(b)** 2D interaction of 3HKT docked with 2-[3-(2-bromophenyl)-1,2,4-oxadiazol-5-yl]cyclopentane-1-carboxylic acid (15b) using AutoDock Tools 1.7, visualized in Discovery Studio: serine 43, glycine 25 and arginine 356 form conventional hydrogen bonds represented in green dotted lines with the ligand. **(c)** 2D interaction of 3HKT docked with 3-[3-(4-bromo-2-methylphenyl)-1,2,4-oxadiazol-5-yl]cyclopentane-1-carboxylic acid (15e) using AutoDock Tools 1.7, visualized in Discovery Studio: residue serine 43, serine 154, proline 24 and arginine 356 form conventional hydrogen bonds represented in green dotted lines with the ligand. **(d)** 3D orientation of 4 potential inhibitors with the 3HKT docked using AutoDock Tools 1.7, the potential inhibitors are represented in ball and stick format. 4-[3-(4-bromophenyl)-1,2,4-oxadiazol-5-yl]butanoic acid in deep blue, 2‐[3‐(4‐bromophenyl)‐1,2‐oxazol‐5‐yl]cyclobutane‐1‐carboxylic acid in magneta, 2-[3-(2-bromophenyl)-1,2,4-oxadiazol-5-yl]cyclopentane-1-carboxylic acid in green, 3-[3-(4-bromo-2-methylphenyl)-1,2,4-oxadiazol-5-yl]cyclopentane-1-carboxylic acid in yellow.

The binding energies of the top three hit compounds are presented in Table 2. The three compounds had binding energies lower than 17122279 suggesting that the compound might be better larvicidal candidates than previously reported compounds. Further evaluation of their 2D interaction reveals that replacing alkyl chain on C-5 with cycloalkyl chain result in a Pi-Alkyl interaction with tyrosine residue 256 of the B chain of *Ag*3HKT (as seen in Fig. 3a-(c)). All compounds formed at least a conventional hydrogen bond with serine 43 on chain B and arginine 356 on chain C. Addition hydrogen bonds were formed with either glycine 25 or serine 154 or proline 24 (Fig. 3a–(c)). All the ligands shared similar 3D orientation with 17122279 (Fig. 3d).

**Table 2:** Final top 3 hits from the docking studies and the co-crystallized ligand with their binding energies and inhibition constants when bound to the *Ag*3HKT protein target (PDB ID: 2CH2).

Pubchem ID	Compound name	Structure	Binding energy, ΔG *(kcal/mol)*	Inhibition Constant, Ki *(μM)*
Cmpd1	2‐[3‐(4‐bromophenyl)‐1,2‐oxazol‐5‐yl]cyclopentane‐1‐ carboxylic acid		−8.58	0.52
103979784 (15b)	2-[3-(4-bromophenyl)-1,2,4-oxadiazol-5-yl]cyclopentane-1-carboxylic acid		−8.25	0.90
103552166 (15e)	3-[3-(4-bromo-2-methylphenyl)-1,2,4-oxadiazol-5-yl]cyclopentane-1-carboxylic acid		−8.18	1.00
6420149 (co-crystallized ligand)	4-(2-aminophenyl)-4-oxobutanoic acid		−6.17	30.15

### 3.2 ADMET screening

ADMET properties of top hits were compared to those of temephos (a standard larvicide), as well as reported 3HKT inhibitors (6420149, 17122279 and its ester derivative). All the potential *Ag*3HKT inhibitors were predicted to be more soluble than temephos by ESOL and Ali classification (Table 3). The permeability coefficient, log Kp (cm/s), predicts the transport of compounds across human epidermis [45]. The more negative its value, the lesser the skin permeability of the compound. All compounds had more negative values than temephos (a known larvicide), suggesting they might penetrate the skin lesser than temephos. However, the co-crystallized compound (6420149) had the most negative value. Brenks filter gives a structural alert of fragments in compounds that likely to be toxic and unstable. All compounds had no structural alert for BRENKS except the co-crystallized ligand (6420149) and temephos. This suggests that all top three compounds are likely to be non-toxic and stable. Similarly, all compounds had no structural for PAINS filter, suggesting that these compounds might be target specific and not promiscuous binders. Synthetic accessibility score which ranges from 1 (very easy) to 10 (very difficult to synthetize) predicts the ease of synthesis. All compounds had synthetic accessibility score <4. Although, the three top compounds had synthetic accessibility score greater than 6420149 and 17122279, these scores were comparable values with that of temephos, this points to them being synthesizable. All three compounds were predicted to be biodegradable with probabilities greater than that of 6420149, 17122279 (the 3HKT inhibitor with lowest LC_50_ in *Ae. aegypti*) and its ester. All potential Ag3HKT inhibitors were predicted to be non-toxic to honeybee while temephos was predicted to be toxic. 103552166 (15e) as well as temephos and ester derivative of 17122279 were predicted to be toxic to crustaceans while other compounds were non-toxic. Temephos, as well as all top three potential *Ag*3HKT inhibitors were predicted to be toxic to fish. 6420149 and 17122279 were predicted to be non-toxic while ester of 17122279 was toxic. All top three compounds were predicted to be safer compared to temephos, a standard larvicide.

**Table 3:** ADMET properties of compounds.

Parameters	6420149	17122279 (6d)	Cmpd1	103979784 (15b)	103552166 (15e)	17122279 ester	Temephos
ESOL Class	Very soluble	Soluble	Moderately soluble	Moderately soluble	Moderately soluble	Soluble	Poorly soluble
Silicos-IT class	Soluble	Moderately soluble	Moderately soluble	Moderately soluble	Moderately soluble	Moderately soluble	Moderately soluble
Ali Class	Soluble	Soluble	Moderately soluble	Moderately soluble	Moderately soluble	Soluble	Poorly soluble
Synthetic Accessibility	1.31	2.7	3.64	3.56	3.76	2.77	3.54
log Kp (cm/s)	−6.73	−6.32	−5.96	−6.13	−6.09	−6.24	−4.91
Honey bee toxicity (probability) - *Toxic*	–	–	–	–	–	–	0.9768
Honey bee toxicity (probability) - *Non-Toxic*	0.9493	0.9653	0.949	0.9512	0.9619	0.9172	–
Biodegradation (probability) - *Degradable*	0.5	0.7	0.75	0.7750	0.825	0.675	0.925
Biodegradation (probability) - *Not Degradable*	–	–	–	–	–	–	–
Crustacea aquatic toxicity (probability) - *Toxic*	–	–	–	–	0.63	0.5245	0.91
Crustacea aquatic toxicity (probability) - *Non-Toxic*	0.87	0.74	0.5252	0.5300	–	–	–
Fish aquatic toxicity (probability) - *Toxic*	–	–	0.9378	0.8999	0.9266	0.6475	0.9928
Fish aquatic toxicity (probability) - *Non-Toxic*	0.4191	0.5606	–	–	–	–	–

6420149: 4-(2-aminophenyl)-4-oxobutanoic acid; **17122279 (6d)**: 4-[3-(4-bromophenyl)-1,2,4-oxadiazol-5-yl]butanoic acid; **Cmpd1**: 2-[3-(4-bromophenyl)-1,2-oxazol-5-yl]cyclopentane-1- carboxylic acid; **103979784(15b)**: 2-[3-(4-bromophenyl)-1,2,4-oxadiazol-5-yl]cyclopentane-1-carboxylic acid; **103552166 (15e)**: 3-[3-(4-bromo-2-methylphenyl)-1,2,4-oxadiazol-5-yl]cyclopentane-1-carboxylic acid; **17122279 ester**: 4-[3-(4-bromophenyl)-1,2,4-oxadiazol-5-yl]butanoic acid; **Temephos**: [4-(4-dimethoxyphosphinothioyloxyphenyl)sulfanylphenoxy]-dimethoxy-sulfanylidene-lambda5-phosphane.

### 3.3 Structural dynamics of HCA upon ligand binding

**Fig. 4:**
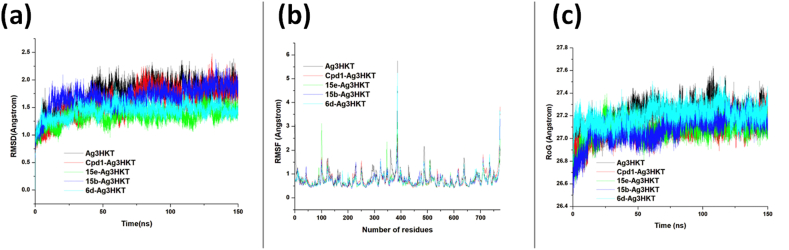
**(a)** Backbone RMSDs are depicted as a function of time for the unbound HCA and bound HCAs. **(b)** Cα fluctuation for unbound HCA and bound HCA. **(c)** Backbone ROG of the unbound HCA and bound HCAs.

From the RMSD plot the systems achieved equilibration at 50 ns, and the stability remained unchanged throughout the simulation period (Fig. 4a). This indicated that the system is stable and could be used for further downstream analyses. While the *Ag*3HKT system had a higher average RMSD of 1.63 Å, Cmpd1 and 15e bound systems had 1.47 Å and 1.41 Å respectively. Similarly, the RMSF plot which is used to investigate the fluctuation of atoms in the course of simulation, revealed that the RMSF of Cmpd1 and 15e bound systems in relative to the *Ag*3HKT system followed similar trend as the RMSD (Fig. 4b). The average RMSF of Cmpd1 and 15e bound systems were 0.73 Å and 0.71 Å respectively while that of the *Ag*3HKT system was 0.80 Å. For the ROG, which is a measure of the compactness of a system showed that the *Ag*3HKT protein exhibited less compactness when compared to the Cmpd1 and 15e bound systems (Fig. 4c). This observation corroborated the findings seen in the RMSD and RMSF plots. Collectively these results showed that Cmpd1 and 15e bound systems exhibited good binding and stabilizing effect on the protein. Taking a cursory look at the behaviour of 15b and 6d bound systems, it is evident that while 15b and 6d showed a measure of stabilizing the system, they however did not exhibit more stabilizing effect on the protein. This further explained the behaviour of 15b and 6d within the active site of the proteins showing a ligand RMSD of 2.37 Å and 2.41 Å respectively, while Cmpd1 and15e had 1.98 Å and 1.74 Å respectively.

### 3.4 Thermodynamics binding of HCA and ligands

The use of MM/PBSA technique to calculate free binding energy could be used to get insight into protein-ligand interaction systems. We explored the time-wise bond interaction between critical residues in the active sites of *Ag*3HKT and Cmpd1, 15e, 15b, and 6d. The thermodynamic calculations showed that the total binding energies (ΔG_bind_) between Cmpd1, 15e and *Ag*3HKT were −26.64 kcal/mol and −24.26 kcal/mol, respectively (Table 4). While that of 15b, 6d upon binding to *Ag*3HKT were −14.11 kcal/mol and −12.02 kcal/mol, respectively. We visualized the 2D interactions in order to further study the contribution of each active site residue to the protein-ligand interactions. Residues within the active sites of the variants elicited various energy type most especially hydrogen bond, others include hydrophobic interaction, and pi-alkyl.

**Table 4:** Binding free energy of *Ag*3HKT upon binding to the corresponding ligand.

Energy Component	Cmpd1- *Ag*3HKT	15e- *Ag*3HKT	15b-*Ag*3HKT	6d- *Ag*3HKT
ΔE_vdW_ (kcal/mol)	−21.22	−20.34	12.08	10.22
ΔE_ele_ (kcal/mol)	−40.64	−41.87	−20.10	−17.65
ΔG_gas_ (kcal/mol)	−65.21	−50.98	−43.12	−27.09
ΔG_sol_ (kcal/mol)	19.08	12.44	18.96	11.84
ΔG_bind_ (kcal/mol)	−26.64	−24.26	−14.11	−12.02

**Cmpd1**: 2-[3-(4-bromophenyl)-1,2-oxazol-5-yl]cyclopentane-1- carboxylic acid; **15e (103552166)**: 3-[3-(4-bromo-2-methylphenyl)-1,2,4-oxadiazol-5-yl]cyclopentane-1-carboxylic acid; **15b (103979784)**: 2-[3-(4-bromophenyl)-1,2,4-oxadiazol-5-yl]cyclopentane-1-carboxylic acid; **6d (17122279)**: 4-[3-(4-bromophenyl)-1,2,4-oxadiazol-5-yl]butanoic acid.

## 4 Discussion

The study employed hybrid virtual screening and molecular dynamics simulation to identify potential inhibitors of *Ag*3HKT which could serve as novel larvicidal compounds. The molecular docking results showed that 2-[3-(4-bromophenyl)-1,2-oxazol-5-yl]cyclopentane-1- carboxylic acid, 2-[3-(4-bromophenyl)-1,2,4-oxadiazol-5-yl]cyclopentane-1-carboxylic acid, 4-[3-(4-bromophenyl)-1,2-oxazol-5-yl]butanoate and 4-(3-(4-bromophenyl)-1,2,4-oxadiazol-5-yl)butanoic acid derivatives having bromine functional group on the C-4 of the phenyl ring had binding energies of −8.58, −8.25, −7.8, −7.38 kcal/mol (Table 1). Thus, the results suggest that cycloalkyl-1-carboxylic acid replacement at the alkyl chain located at C-5 of isoxazole or 1,2,4-oxadiazole nucleus yield compounds with better binding affinity than their straight chain counterparts, with isoxazoles having higher binding affinity than their 1,2,4-oxadiazole counterparts. Sapko et al. [49] reported (+)-(1S,2S)-2-(3,4-dichlorobenzoyl)-cyclopropyl-1-carboxylic acid (DBCC) as an HKT inhibitor, featuring a cyclopropyl linker and a carboxylic acid end [49]. We examined the binding affinity of this compound in the *Ag*3HKT binding pocket for comparison with our compounds. It yielded a binding energy of −7.8 kcal/mol, which was higher than the binding energies of our best hits from the virtual screening. The binding interaction of DBCC with *Ag*3HKT (PDB ID: 2CH2) showed conventional hydrogen bonds with asparagine 44, glutamine 204, lysine 205, and threonine 259 via the carboxylic acid end linked to the cyclopropyl. Additionally, a similar pi-alkyl interaction was observed for the best hit from the virtual screening (Cmpd1) with the tyrosine residue 256 of the B chain of *Ag*3HKT. This interaction was also noted between the aromatic ring of DBCC and the tyrosine residue (Fig. S1).

The top compounds from the virtual screening were 2‐[3‐(4‐bromophenyl)‐1,2‐oxazol‐5‐yl]cyclopentane‐1-carboxylic acid (Cmpd1), 2-[3-(4-bromophenyl)-1,2,4-oxadiazol-5-yl]cyclopentane-1-carboxylic acid (15b) and 3-[3-(4-bromo-2-methylphenyl)-1,2,4-oxadiazol-5-yl]cyclopentane-1-carboxylic acid (15e) had binding energies of 8.58, −8.25 and 8.18 kcal/mol, respectively (Table 2). The best hit is an isoxazole, while the other top 2 hits are oxadiazoles. These groups of heterocyclic compounds form important frameworks for many marketed drugs and have immense potential for exploration [50]. The three best hits were predicted to be easy to synthesize, as indicated by the SWISSADME webserver. This prediction aligns with the recent reported opportunities for one-pot and room temperature synthesis for these heterocycles [51]. These compounds were also predicted to be safer compared to temephos, a standard larvicide used. The top three compounds reported in this study differed in binding energy from 4-(2-aminophenyl)-4-oxobutanoic acid, the co-crystallized compound with greater than 1.433 kcal/mol. However, the compounds did not differ from each other or from compound 17122279 by < 6 kJ/mol. Genheden and Ryde [52] reported that molecular docking might be inefficient in discriminating between compounds which differ by < 6 kJ/mol (1.433 kcal/mol). Hence, further analysis to assess the affinities of these three best hits for *Ag*3HKT compared to compound 17122279 using MD simulation was carried out.

The study evaluated the stability and binding properties of various ligand bound systems of *Ag*3HKT protein using RMSD, RMSF, and ROG plots and MM/PBSA calculations. The results showed that Cmpd1 and 15e bound systems exhibited good stability and binding properties with average RMSD of 1.47 Å and 1.41 Å and average RMSF of 0.73 Å and 0.71 Å, respectively. The total binding energies between Cmpd1 and 15e and *Ag*3HKT were found to be −26.64 kcal/mol and −24.26 kcal/mol, respectively, while 15b and 6d had lower binding energies of −14.11 kcal/mol and −12.02 kcal/mol respectively. A 2D interaction analysis showed that the active site residues contributed to protein-ligand interactions through hydrogen bonds, hydrophobic interactions, and pi-alkyl. The stability of the systems was found to be adequate for further downstream analyses and the results showed that the systems reached equilibration at 50 ns. The use of RMSD and RMSF plots, along with the ROG measure of compactness, gave an overall picture of the stability and binding properties of the systems. The MM/PBSA calculations helped to understand the binding energy contributions and the interaction between the critical residues in the active site.

The virtual result in this study corroborates the finding of previous studies in which 17122279 was reported to be a better inhibitor than *Ag*3HKT co-crystallized inhibitor [14,15,26]. All isoxazole compounds tested in this current study had lower binding energy than their oxadiazole counterparts, with butane/cyclobutane derivatives having lower binding energy than their propane/cyclopropane counterparts (Table 1). This supports the report from a previous study [26], in which replacement of oxadiazole nucleus with an isoxazole, as well as increasing alkyl chain length was reported to yield better larvicidal candidates. A quick trial on the effect of replacing with a cyclohexane resulted in a binding energy of −9.1 kcal/mol. This current study reveals the impact of replacing the alkyl chain on C5 of isoxazole or oxadiazole derivatives with a cycloalkyl, as well as increasing chain length (pentane or hexane) on the binding energy of these compounds for *Ag*3HKT.

While previous studies have focused on modulation of attachment on the C-3 of the isoxazole or oxadiazole derivatives as well as chain length on C-5, this present study suggests that additional modification on the C-5 atom of these nucleus (by replacing alkyl chain with cycloalkyl chain) might yield better larvicidal candidates. This study employed molecular dynamic simulation in addition to molecular docking as compared to other studies on 3HKT inhibitors. This strategy offers an advantage of providing insight about the stability of the ligand-receptor complex. However, synthesis and experimental testing of predicted compounds remains to be done.

## 5 Conclusion

This study successfully employed a combination of hybrid virtual screening and molecular dynamics simulations to identify novel potential inhibitors of *Ag*3HKT, which could serve as promising larvicidal agents. Molecular docking results indicated that these compounds, characterized by the cycloalkyl-1-carboxylic acid substitution at the C-5 position, exhibited better binding energies and favorable interactions within the active site of Ag3HKT. Additionally, the molecular dynamics simulations showed that Cmpd1 and 15e maintained stable ligand-protein complexes throughout the simulations, with low RMSD and RMSF values, further corroborating their strong binding and stable interactions. The MM/PBSA calculations also revealed that these compounds have better binding free energies compared to other derivatives, further validating their potential as effective inhibitors.

Furthermore, these top compounds were predicted to be easy to synthesize and exhibited safety profiles compared to the standard larvicide temephos, offering a promising pathway for developing more efficient and safer larvicidal agents. The findings also provide valuable insights into the importance of structural modifications, such as the introduction of cycloalkyl chains at key positions, which significantly enhance binding affinity. While the *in silico* findings are promising, experimental synthesis and testing are needed to validate the efficacy of these compounds.

## CRediT authorship contribution statement

**Eunice O. Adedeji:** Writing – review & editing, Writing – original draft, Visualization, Validation, Software, Methodology, Investigation, Formal analysis, Data curation, Conceptualization. **Olubanke O. Ogunlana:** Writing – review & editing, Supervision, Project administration. **Gbolahan O. Oduselu:** Writing – review & editing, Formal analysis, Conceptualization. **Rainer Koenig:** Writing – review & editing, Supervision, Project administration, Funding acquisition. **Ezekiel Adebiyi:** Writing – review & editing, Supervision, Project administration, Funding acquisition. **Opeyemi S. Soremekun:** Writing – review & editing, Writing – original draft, Visualization, Validation, Software, Methodology, Investigation, Formal analysis, Data curation, Conceptualization. **Segun Fatumo:** Writing – review & editing, Supervision, Resources, Project administration, Funding acquisition, Conceptualization.

## Ethics and consent declarations

Review and/or approval by an ethics committee as well as informed consent was not required for this study because this article did not involve any direct experimentation/studies on living beings.

## Data availability statement

Data is included in article/supplementary material/referenced in article.

## Funding

This work was supported by 10.13039/501100001659Deutsche Forschungsgemeinschaft (DFG) [grant number KO 3678/5-1]; 10.13039/100000061Fogarty 10.13039/100000002National Institutes of Health (NIH) Common Fund [grant number U2RTW010679]. SF was supported by Wellcome Trust [grant number 220740/Z/20/Z]. The funders had no role in the design of the study; in the collection, analyses, or interpretation of data; in the writing of the manuscript; or in the decision to publish the results.

## Declaration of competing interest

The authors declare the following financial interests/personal relationships which may be considered as potential competing interests: SF is an AE of Heliyon journal. If there are other authors, they declare that they have no known competing financial interests or personal relationships that could have appeared to influence the work reported in this paper.

## Figures and Tables

**Figure:**
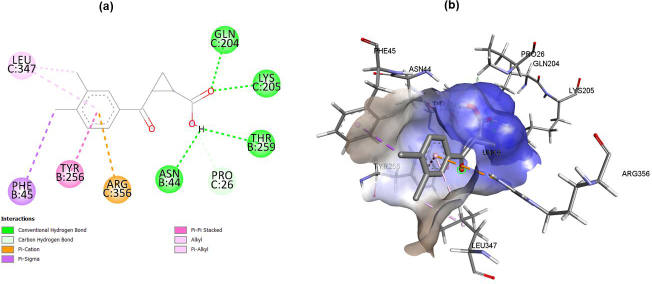
(a) 2D interaction of 3HKT docked with (+)-(1S,2S)-2-(3,4-dichlorobenzoyl)-cyclopropyl-1-carboxylic acid (DBCC): residue asparagine 44, glutamine 204, lysine 205, threonine 259 form conventional hydrogen bonds represented in green dotted lines with the ligand. (b) 3D orientation of (+)-(1S,2S)-2-(3,4-dichlorobenzoyl)-cyclopropyl-1-carboxylic (DBCC) docked with 3HK, showing the hydrophobic surface.

## References

[1] World Health Organisation (2023).

[2] Finney M., McKenzie B.A., Rabaovola B., Sutcliffe A., Dotson E., Zohdy S. (2021). Widespread zoophagy and detection of Plasmodium spp. in Anopheles mosquitoes in southeastern Madagascar. Malar. J..

[3] Singh M., Singh G., Dubey A., Chaitanya R. (2021). *Plasmodium*'s journey through the *Anopheles* mosquito: a comprehensive review. Biochimie.

[4] Sinka M.E., Bangs M.J., Manguin S., Coetzee M., Mbogo C.M., Hemingway J., Patil A.P., Temperley W.H., Gething P.W., Kabaria C.W. (2010). The dominant Anopheles vectors of human malaria in Africa, Europe and the Middle East: occurrence data, distribution maps and bionomic précis. Parasit Vectors.

[5] World Health Organization (2022). Manual for monitoring insecticide resistance in mosquito vectors and selecting appropriate interventions.

[6] Dara D., Drabovich A.P. (2022). Assessment of risks, implications, and opportunities of waterborne neurotoxic pesticides. Journal of Environmental Sciences.

[7] Kouadio F.-P.A., Wipf N.C., Nygble A.S., Fodjo B.K., Sadia C.G., Vontas J., Mavridis K., Müller P., Mouhamadou C.S. (2023). Relationship between insecticide resistance profiles in Anopheles gambiae sensu lato and agricultural practices in Côte d'Ivoire. Parasit Vectors.

[8] Mawejje H.D., Weetman D., Epstein A., Lynd A., Opigo J., Maiteki-Sebuguzi C., Lines J., Kamya M.R., Rosenthal P.J., Donnelly M.J. (2023). Characterizing pyrethroid resistance and mechanisms in Anopheles gambiae (ss) and Anopheles arabiensis from 11 districts in Uganda. Current Research in Parasitology & Vector-Borne Diseases.

[9] Yusuf M.A., Oshaghi M.A., Vatandoost H., Hanafi-Bojd A.A., Enayati A., Jalo R.I., Aminu A.O.A.A., Daneji I.M. (2021). Current status of insecticide susceptibility in the principal malaria vector, *Anopheles gambiae* in three northern states of Nigeria. Journal of arthropod-borne diseases.

[10] Tepa A., Kengne-Ouafo J.A., Djova V.S., Tchouakui M., Mugenzi L.M., Djouaka R., Pieme C.A., Wondji C.S. (2022). Molecular Drivers of Multiple and Elevated Resistance to Insecticides in a Population of the Malaria Vector Anopheles gambiae in Agriculture Hotspot of West Cameroon. Genes.

[11] Pires S., Alves J., Dia I., Gómez L.F. (2020). Susceptibility of mosquito vectors of the city of Praia, Cabo Verde, to Temephos and Bacillus thuringiensis var israelensis. PLoS One.

[12] Sparks T.C., Storer N., Porter A., Slater R., Nauen R. (2021). Insecticide resistance management and industry: the origins and evolution of the I nsecticide Resistance Action Committee (IRAC) and the mode of action classification scheme. Pest Manage. Sci..

[13] Duke S.O., Pan Z., Chittiboyina A.G., Swale D.R., Sparks T.C. (2023). Molecular targets of insecticides and herbicides–Are there useful overlaps?. Pestic. Biochem. Physiol..

[14] Maciel L.G., Barbosa A.d.S., de Alencar-Filho E.B., Soares T.A., Dos Anjos J.V. (2021). A second generation of 1, 2, 4-oxadiazole derivatives with enhanced solubility for inhibition of 3-hydroxykynurenine transaminase (HKT) from *Aedes aegypti*. RSC Med. Chem..

[15] Maciel L.G., Oliveira A.A., Romão T.P., Leal L.L., Guido R.V., Silva-Filha M.H.N., dos Anjos J.V., Soares T.A. (2020). Discovery of 1, 2, 4-oxadiazole derivatives as a novel class of noncompetitive inhibitors of 3-hydroxykynurenine transaminase (HKT) from *Aedes aegypti*. Biorg. Med. Chem..

[16] Rossi F., Lombardo F., Paglino A., Cassani C., Miglio G., Arcà B., Rizzi M. (2005). Identification and biochemical characterization of the *Anopheles gambiae* 3-hydroxykynurenine transaminase. FEBS J..

[17] Adedeji E.O., Ogunlana O.O., Fatumo S., Aromolaran O.T., Beder T., Koenig R., Adebiyi E. (2023). The central metabolism model of Anopheles gambiae: a tool for understanding malaria vector biology. Biotechnological Approaches to Sustainable Development Goals.

[18] Fei X., Zhang Y., Ding L., Xiao S., Xie X., Li Y., Deng X. (2021). Development of an RNAi-based microalgal larvicide for the control of *Aedes aegypti*. Parasit Vectors.

[19] Okuda S., Nishiyama N., Saito H., Katsuki H. (1998). 3‐Hydroxykynurenine, an endogenous oxidative stress generator, causes neuronal cell death with apoptotic features and region selectivity. J. Neurochem..

[20] Colín-González A.L., Maldonado P.D., Santamaría A. (2013). 3-Hydroxykynurenine: an intriguing molecule exerting dual actions in the central nervous system. Neurotoxicology.

[21] Han Q., Fang J., Li J. (2002). 3-hydroxykynurenine transaminase identity with alanine glyoxylate transaminase a probable detoxification protein in *Aedes aegypti*. J. Biol. Chem..

[22] Han Q., Beerntsen B.T., Li J. (2007). The tryptophan oxidation pathway in mosquitoes with emphasis on xanthurenic acid biosynthesis. J. Insect Physiol..

[23] Rossi F., Garavaglia S., Giovenzana G.B., Arcà B., Li J., Rizzi M. (2006). Crystal structure of the *Anopheles gambiae* 3-hydroxykynurenine transaminase. Proc. Natl. Acad. Sci. USA.

[24] Canavesi R., Miggiano R., Stella M., Galli U., Rossi F., Rizzi M., Del Grosso E. (2019). Study of *Anopheles gambiae* 3-hydroxykynurenine transaminase activity and inhibition by LC-MS/MS method. J. Pharmaceut. Biomed. Anal..

[25] da Silva-Alves D.C., dos Anjos J.V., Cavalcante N.N., Santos G.K., do Af Navarro D.M., Srivastava R.M. (2013). Larvicidal isoxazoles: synthesis and their effective susceptibility towards *Aedes aegypti* larvae. Biorg. Med. Chem..

[26] Oliveira V.S., Pimenteira C., da Silva-Alves D.C., Leal L.L., Neves-Filho R.A., Navarro D.M., Santos G.K., Dutra K.A., dos Anjos J.V., Soares T.A. (2013). The enzyme 3-hydroxykynurenine transaminase as potential target for 1, 2, 4-oxadiazoles with larvicide activity against the dengue vector *Aedes aegypti*. Biorg. Med. Chem..

[27] Neves Filho R.A.W., da Silva C.A., da Silva C.S.B., Brustein V.P., Navarro D.M.d.A.F., dos Santos F.A.B., Alves L.C., dos Santos Cavalcanti M.G., Srivastava R.M., das Graças Carneiro-Da-Cunha M. (2009). Improved microwave-mediated synthesis of 3-(3-aryl-1, 2, 4-oxadiazol-5-yl) propionic acids and their larvicidal and fungal growth inhibitory properties. Chem. Pharm. Bull..

[28] Zhao Z., Li R., Li Y. (2014). Highly efficient and clean synthesis of 1-amino-2-acetylanthraquinone by copper-catalyzed reductive cleavage of isoxazole motif. Chin. J. Catal..

[29] Maciel L.G., Ferraz M.V., Oliveira A.A., Lins R.D., Dos Anjos J.V., Guido R.V., Soares T.A. (2023). Inhibition of 3-Hydroxykynurenine Transaminase from Aedes aegypti and Anopheles gambiae: a mosquito-specific target to combat the transmission of arboviruses. ACS bio & med Chem Au.

[30] Burley S.K., Bhikadiya C., Bi C., Bittrich S., Chen L., Crichlow G.V., Christie C.H., Dalenberg K., Di Costanzo L., Duarte J.M. (2021). RCSB Protein Data Bank: powerful new tools for exploring 3D structures of biological macromolecules for basic and applied research and education in fundamental biology, biomedicine, biotechnology, bioengineering and energy sciences. Nucleic Acids Res..

[31] Morris G.M., Huey R., Lindstrom W., Sanner M.F., Belew R.K., Goodsell D.S., Olson A.J. (2009). AutoDock4 and AutoDockTools4: automated docking with selective receptor flexibility. J. Comput. Chem..

[32] Huey R., Morris G.M., Forli S. (2012).

[33] Kim S., Chen J., Cheng T., Gindulyte A., He J., He S., Li Q., Shoemaker B.A., Thiessen P.A., Yu B. (2021). PubChem in 2021: new data content and improved web interfaces. Nucleic Acids Res..

[34] Lipinski C.A. (2004). Lead-and drug-like compounds: the rule-of-five revolution. Drug Discov. Today Technol..

[35] Rappé A.K., Casewit C.J., Colwell K., Goddard W.A., Skiff W.M. (1992). UFF, a full periodic table force field for molecular mechanics and molecular dynamics simulations. J. Am. Chem. Soc..

[36] Dallakyan S., Olson A.J. (2015). Small-molecule library screening by docking with PyRx. Chem. Biol..

[37] O'Boyle N.M., Banck M., James C.A., Morley C., Vandermeersch T., Hutchison G.R. (2011). Open Babel: an open chemical toolbox. J. Cheminform..

[38] Axon Chem (2022). MarvinSketch Vers. 22.11.

[39] Trott O., Olson A.J. (2010). AutoDock Vina: improving the speed and accuracy of docking with a new scoring function, efficient optimization, and multithreading. J. Comput. Chem..

[40] Morris G.M., Goodsell D.S., Halliday R.S., Huey R., Hart W.E., Belew R.K., Olson A.J. (1998). Automated docking using a Lamarckian genetic algorithm and an empirical binding free energy function. J. Comput. Chem..

[41] BIOVIA Dassault Systèmes (2022).

[42] Pettersen E.F., Goddard T.D., Huang C.C., Couch G.S., Greenblatt D.M., Meng E.C., Ferrin T.E. (2004). UCSF Chimera—a visualization system for exploratory research and analysis. J. Comput. Chem..

[43] Daina A., Michielin O., Zoete V. (2017). SwissADME: a free web tool to evaluate pharmacokinetics, drug-likeness and medicinal chemistry friendliness of small molecules. Sci. Rep..

[44] Yang H., Lou C., Sun L., Li J., Cai Y., Wang Z., Li W., Liu G., Tang Y. (2019). admetSAR 2.0: web-service for prediction and optimization of chemical ADMET properties. Bioinformatics.

[45] Potts R.O., Guy R.H. (1992). Predicting skin permeability. Pharm. Res. (N. Y.).

[46] Yang Z., Lasker K., Schneidman-Duhovny D., Webb B., Huang C.C., Pettersen E.F., Goddard T.D., Meng E.C., Sali A., Ferrin T.E. (2012). UCSF Chimera, MODELLER, and IMP: an integrated modeling system. J. Struct. Biol..

[47] Wang J., Wolf R.M., Caldwell J.W., Kollman P.A., Case D.A. (2004). Development and testing of a general amber force field. J. Comput. Chem..

[48] Kollman P.A., Massova I., Reyes C., Kuhn B., Huo S., Chong L., Lee M., Lee T., Duan Y., Wang W., Donini O., Cieplak P., Srinivasan J., Case D.A., Cheatham T.E. (2000). Calculating structures and free energies of complex molecules: combining molecular mechanics and continuum models. Acc. Chem. Res..

[49] Sapko M.T., Guidetti P., Yu P., Tagle D.A., Pellicciari R., Schwarcz R. (2006). Endogenous kynurenate controls the vulnerability of striatal neurons to quinolinate: implications for Huntington's disease. Exp. Neurol..

[50] Thakur A., Verma M., Bharti R., Sharma R. (2022). Oxazole and isoxazole: from one-pot synthesis to medical applications. Tetrahedron.

[51] Baykov S.V., Shetnev A.A., Semenov A.V., Baykova S.O., Boyarskiy V.P. (2023). Room temperature synthesis of bioactive 1,2,4-oxadiazoles. Int. J. Mol. Sci..

[52] Genheden S., Ryde U. (2015). The MM/PBSA and MM/GBSA methods to estimate ligand-binding affinities. Expet Opin. Drug Discov..

