# Supplementary material for: Molecular docking and molecular dynamics simulation studies of inhibitor candidates against Anopheles gambiae 3-hydroxykynurenine transaminase and implications on vector control
Source: Heliyon. 2025 Jan 2;11(1):e41633. doi: 10.1016/j.heliyon.2025.e41633 (PMC11759636; doi:10.1016/j.heliyon.2025.e41633)
Supplement: Multimedia component 1 [file mmc1.docx]

**Table S1:** Binding energies of the 50 best hits from the virtual screening of the 958 compounds against *Ag*3HKT (PDB ID: 2CH2)

| **S/N** | **PubChem ID/ Compound ID** | **SMILES** | **Binding energy (kcal/mol)** |
| --- | --- | --- | --- |
| 1 | Cpd1 | O=C(C1C(C2=CC(C3=CC=C(Br)C=C3)=NO2)CCC1)O | -8.58 |
| 2 | 103979784 | C1CC(C(C1)C(=O)O)C2=NC(=NO2)C3=CC=C(C=C3)Br | -8.25 |
| 3 | 103552166 | CC1=C(C=CC(=C1)Br)C2=NOC(=N2)C3CCC(C3)C(=O)O | -8.18 |
| 4 | 43514032 | C1CCC(C(C1)C2=NC(=NO2)C3=CC=C(C=C3)Br)C(=O)O | -8.12 |
| 5 | 104963035 | C1C=CCC(C1C2=NC(=NO2)C3=CC=CC=C3Br)C(=O)O | -8.10 |
| 6 | 43150830 | C1C=CCC(C1C2=NC(=NO2)C3=CC(=CC=C3)Br)C(=O)O | -8.10 |
| 7 | 103979806 | C1CC(C(C1)C(=O)O)C2=NC(=NO2)C3=CC=CC=C3Br | -8.00 |
| 8 | 43514031 | C1C=CCC(C1C2=NC(=NO2)C3=CC=C(C=C3)Br)C(=O)O | -8.00 |
| 9 | 103552002 | CC1=CC(=CC=C1)C2=NOC(=N2)C3CCC(C3)C(=O)O | -7.90 |
| 10 | 103552029 | CC1=C(C=CC(=C1)C2=NOC(=N2)C3CCC(C3)C(=O)O)F | -7.90 |
| 11 | 165351297 | C1=CC=C(C=C1)C2=NOC(=N2)C(C(C(C(=O)O)(F)F)(F)F)(F)F | -7.90 |
| 12 | 29081591 | C1C=CCC(C1C2=NC(=NO2)C3=CC(=CC=C3)Br)C(=O)O | -7.90 |
| 13 | 103500709 | CC1=CC(=CC(=C1Br)C)C2=NOC(=N2)C(C)C(C)C(=O)O | -7.80 |
| 14 | 103551999 | CC1=CC=C(C=C1)C2=NOC(=N2)C3CCC(C3)C(=O)O | -7.80 |
| 15 | 103552026 | C1CC(CC1C2=NC(=NO2)C3=CC(=C(C=C3)F)F)C(=O)O | -7.80 |
| 16 | 103551999 | CC1=CC=C(C=C1)C2=NOC(=N2)C3CCC(C3)C(=O)O | -7.80 |
| 17 | 103552026 | C1CC(CC1C2=NC(=NO2)C3=CC(=C(C=C3)F)F)C(=O)O | -7.80 |
| 18 | 103552037 | C1CC(CC1C2=NC(=NO2)C3=CC(=CC(=C3)F)F)C(=O)O | -7.80 |
| 19 | 61724599 | C1C=CCC(C1C2=NC(=NO2)C3=CC=CC=C3Br)C(=O)O | -7.80 |
| 20 | 103551999 | CC1=CC=C(C=C1)C2=NOC(=N2)C3CCC(C3)C(=O)O | -7.80 |
| 21 | 103552053 | C1CC(CC1C2=NC(=NO2)C3=CC=CC=C3F)C(=O)O | -7.80 |
| 22 | 103552115 | CC1=CC(=CC(=C1)F)C2=NOC(=N2)C3CCC(C3)C(=O)O | -7.70 |
| 23 | 103551992 | CC1=C(C=C(C=C1)C2=NOC(=N2)C3CCC(C3)C(=O)O)F | -7.60 |
| 24 | 103552024 | CCC1=CC=C(C=C1)C2=NOC(=N2)C3CCC(C3)C(=O)O | -7.60 |
| 25 | 103979779 | C1CC(C(C1)C(=O)O)C2=NC(=NO2)C3=CC(=CC=C3)Br | -7.60 |
| 26 | 114392635 | CC1CC(C(C1)C(=O)O)C2=NC(=NO2)C3=CC=C(C=C3)Br | -7.60 |
| 27 | 43471110 | C1CCC(C1)(CC2=NC(=NO2)C3=CC(=CC=C3)Br)C(=O)O | -7.60 |
| 28 | 103552005 | C1CC(CC1C2=NC(=NO2)C3=CC=CC=C3)C(=O)O | -7.50 |
| 29 | 103552018 | C1CC(CC1C2=NC(=NO2)C3=CC=C(C=C3)F)C(=O)O | -7.50 |
| 30 | 103552020 | C1CC(CC1C2=NC(=NO2)C3=CC(=CC=C3)F)C(=O)O | -7.50 |
| 31 | 103979784 | C1CC(C(C1)C(=O)O)C2=NC(=NO2)C3=CC=C(C=C3)Br | -7.50 |
| 32 | 54933651 | CC1=C(C=CC(=C1)C2=NOC(=N2)C3CC3C(=O)O)F | -7.50 |
| 33 | 60791864 | C1C(C1C(=O)O)C2=NC(=NO2)C3=CC(=CC=C3)C(F)(F)F | -7.50 |
| 34 | 62716404 | C1=CC=C2C(=C1)C=CC=C2C3=NOC(=N3)CCCC(=O)O | -7.50 |
| 35 | 163545294 | C1=CC=C(C(=C1)C2=CC=C(C=C2)C(=O)CCC(=O)O)N | -7.50 |
| 36 | 54918800 | CC1=C(C=C(C=C1)C2=NOC(=N2)C3CC3C(=O)O)F | -7.40 |
| 37 | 61724696 | C1CCC(C1)(CC2=NC(=NO2)C3=CC=CC=C3Br)C(=O)O | -7.40 |
| 38 | 79191524 | C1=C(C=C(C=C1F)F)C2=NOC(=N2)CCCCO | -7.40 |
| 39 | 89457408 | C1=CC=C(C=C1)C=CC(=O)OC(=O)C2=C(C(=CC=C2)O)N | -7.40 |
| 40 | 17122279 | C1=CC(=CC=C1C2=NOC(=N2)CCCC(=O)O)Br | -7.38 |
| 41 | 105489000 | C1=CC=C(C(=C1)C(=O)C2=C(C(=CC=C2)O)N)F | -7.30 |
| 42 | 52996931 | CC1(C(C1C(=O)O)C2=NC(=NO2)C3=CC=CC=C3)C | -7.30 |
| 43 | 61650172 | C1C(C1C(=O)O)C2=NC(=NO2)C3=CC(=C(C(=C3)F)F)F | -7.30 |
| 44 | 69789503 | C1=CC=C(C(=C1)C(=O)C2=C(C(=CC=C2)O)N)N | -7.30 |
| 45 | 104963025 | C1C=CCC(C1C2=NC(=NO2)C3=CC=C(C=C3)Br)C(=O)O | -7.20 |
| 46 | 61069482 | C1CCC(C1)(CC2=NC(=NO2)C3=CC=CC=C3)CC(=O)O | -7.20 |
| 47 | 61648722 | C1C(C1C(=O)O)C2=NC(=NO2)C3=CC(=CC(=C3)F)F | -7.20 |
| 48 | 62934394 | CC1=C(C=C(C=C1)C2=NOC(=N2)CC(C)(C)CC(=O)O)F | -7.20 |
| 49 | 62942393 | CC(C)C1=CC=C(C=C1)C2=NOC(=N2)CC(C)CC(=O)O | -7.20 |
| 50 | 64835382 | CC1=CC(=CC=C1)C2=NOC(=N2)C3C(C3(C)C)C(=O)O | -7.20 |
